# How maternal investment varies with environmental factors and the age and physiological state of wild tsetse *Glossina pallidipes* and *Glossina morsitans morsitans*

**DOI:** 10.1098/rsos.171739

**Published:** 2018-02-14

**Authors:** John W. Hargrove, M. Odwell Muzari, Sinead English

**Affiliations:** 1 Centre of Excellence in Epidemiological Modelling and Analysis (SACEMA), Stellenbosch University, Stellenbosch, South Africa; 2 Tropical Public Health Services, Queensland Health, Cairns, Australia; 3 Behavioural Ecology Group, Department of Zoology, University of Cambridge, Cambridge, UK

**Keywords:** *Glossina pallidipes*, maternal effects, nutrition, pregnancy, tsetse, trade-offs

## Abstract

Theory suggests females should optimize resource allocation across reproductive bouts to maximize lifetime reproduction, balancing current and future reproductive efforts according to physiological state and projected survival and reproduction. Tests of these ideas focus on long-lived vertebrates: few measure age-related reproductive output in iteroparous invertebrates, or partition reserves between those allocated to offspring versus mothers. We investigated how maternal age, and environmental and physiological factors influence reproductive investment in wild tsetse, *Glossina pallidipes* Austen and *G. morsitans morsitans* Westwood. Tsetse provide a tractable system to measure reproductive allocation. Females exhibit high maternal investment, producing single, large offspring that rely exclusively on maternal reserves. We find that mothers in better physiological condition and experiencing cooler temperatures produce larger offspring. Pupal size increases significantly but weakly with age. In both species, females with less fat invest proportionately more in offspring. Post-partum fat decreases in flies with badly frayed wings: poor flight capability may limit their feeding efficiency, or they may sacrifice more reserves as a terminal investment. Our results support evidence that offspring size increases with maternal size, investment depends on the environment, and females with lower chances of future reproduction invest more into current offspring. We discuss the implications of maternal effects for predicting vector population responses to environmental change.

## 1. Introduction

Females that produce several offspring over the course of their lives face a fundamental trade-off: how much of their resources should they invest in current reproduction and how much should they retain for their own survival and for future reproductive opportunities [1–4]? Several theoretical models have been developed to predict how a breeding female should allocate resources to each reproductive attempt across her lifespan: resource allocation strategies will be influenced by her risk of death before breeding again, her physiological condition and the energetic cost of producing offspring [5–7]. Empirical studies largely support these theoretical predictions. To date, these studies have mainly focused on long-lived vertebrate populations (e.g. [8–10]).

There is a growing number of studies investigating how the allocation of resources to reproduction by females varies with age and physiological state in iteroparous invertebrates [11–15]. These studies have, however, mainly been limited to laboratory systems and do not necessarily reflect how females might allocate reserves in the wild, where they face higher mortality risk and more severe physiological stress. Moreover, most studies consider absolute maternal investment (for example, the size of offspring produced), as opposed to relative maternal investment (for example, the proportion of biomass or nutrients transferred from mother to young). As older females tend to have greater access to resources [16], understanding age-related changes in reproductive effort requires consideration of absolute and relative investment. What is required, therefore, are studies which explicitly partition reserves between those transferred to offspring and those kept back for maternal maintenance, particularly in wild invertebrate systems where females breed at multiple points across their lifespan.

Viviparous tsetse flies (*Glossina* species), vectors of the trypanosomes causing nagana in livestock and sleeping sickness in humans [17], present a valuable test of age-related reproductive allocation theory because they are relatively long-lived and exhibit high maternal investment. They produce, at each birth event, a single offspring, which develops into a full-sized adult with no post-birth feeding and no care from either parent. The level of parental care provided for each offspring by its mother is thus completely measured by the amount of fat and protein the female transfers to its larva. Taken together, this makes them nearly unique among metazoan animals. In terms of paternal investment, there is no known interaction between male flies and pupae, and the only known interaction between males and females occurs during copulation—which, for most females, occurs only once in her life. The male's contribution to parental investment seems therefore to be limited to the sperm, and other components of the seminal fluid [18], that he transfers to the female during mating.

The single larva produced is similar in weight to its mother and has 1.5 times her fat content [19]. A female can produce a dozen or more offspring over the course of her life. Thus, females should be adapted to optimize transfer of resources to each individual offspring to maximize total reproductive output across their lifespan. A recent study on *Glossina morsitans morsitans* Westwood in field conditions found that maternal physiological condition and the environment experienced by the female during pregnancy correlate with offspring size according to predictions based on theory [20]. Such patterns have been shown in several other invertebrate systems [21–23]. Specifically, larger females—and those with more fat—produce larger pupae and, as temperatures increase, females become more nutritionally stressed and produce smaller pupae. The authors found no strong indication that females adjust their investment with age, however, although females reproducing for the first time tend to produce relatively smaller offspring.

Here, we expand on this earlier study. We investigate how the size of offspring produced varies with maternal age, physiological condition and temperature in *G. pallidipes* Austen, comparing the results with those previously published for *G*. *m*. *morsitans* captured at the same study site in northern Zimbabwe. *G. pallidipes* are considerably larger than *G. m. morsitans* and the two species differ in their behaviour, with *G. pallidipes* being more likely than *G. m. morsitans* to approach stationary baits, such as odour-baited traps, and relatively less likely to approach mobile baits, such as oxen accompanied by men [24]. At the time of this study, *G. pallidipes* were found at higher population densities in the study area [25,26]. It is unclear how all of these differences will have an impact on patterns of maternal investment. For *G. m. morsitans* we found a weak trend, suggesting that investment tends to increase with age. We have a larger sample size for *G. pallidipes* and thus greater statistical power to investigate this finding further. We also consider relative maternal investment in both species by comparing the proportion of fat in offspring relative to the total fat in mother and offspring at the point of birth. As there is a strong link between size and survival under field conditions of newly emerged flies [27], understanding how mothers influence offspring size and quality, both in terms of common patterns across *G. pallidipes* and *G. m. morsitans* and the contrasts between them, will be an important component of models of tsetse population dynamics and the epidemiology of tsetse-borne disease.

### 1.1. Aims of the study

Our primary interest concerns how the size of *G. pallidipes* offspring produced varies according to female age, physiological state and environmental conditions. We first needed to take into account the fact that older females, captured during warmer months, were themselves born at cooler times of the year, when emerging flies are larger [28]. We tested, therefore, for a correlation between age and size of females, before considering the effects of female age, size, condition, nutritional status and temperature on the size of the offspring she had just produced. We predicted that females in poor physiological condition might invest relatively less in their offspring and more in their own maintenance. At the same time, as females get older and are less likely to survive to produce future offspring, maternal investment in current offspring should increase. We also expected, as in *G. m. morsitans* [20], that females experiencing stressful environmental conditions, during pregnancy (e.g. high temperature), would allocate fewer nutrients to their current offspring. Finally, we considered female costs of reproduction and the trade-off between current and future investment. To this end, we measured how maternal post-partum fat levels varied with maternal age, size and nutrition. Then we used physiological measures and a predictive model of pupal fat in both *G. pallidipes* and *G. m. morsitans* to examine how the relative amount of fat in offspring varied with the total fat in the mother–offspring pair.

## 2. Material and methods

### 2.1. Captures from artificial warthog burrows

Between 5 September and 25 November 1998, and 7 September and 19 November 1999, artificial warthog burrow traps were used to sample adult flies and their offspring at Rekomitjie Research Station, Zambezi Valley, Zimbabwe (16°10' S, 29°25' E, altitude approx. 520 m.a.s.l.). During these months, when mean monthly temperatures range between about 33 and 36°C, female tsetse deposit larvae in subterranean cavities, such as the burrows used by warthogs. Muzari & Hargrove [29] and Hargrove & Muzari [19,30] describe the construction of artificial warthog burrows to simulate these conditions, and the retaining cages used to catch tsetse entering the burrows. Flies were removed from the cages at *ca* 11.15, 12.30, 14.00 and 16.45 and transferred to individually labelled (75 × 25) mm glass tubes. The tubes were placed under a black cloth in a polystyrene box to reduce activity and wing damage. For the purposes of estimating reproductive investment, we were interested in only two classes of flies: (i) post-partum females: those that produced, after capture, a larva that could be attributed unequivocally to that female; this happened when a larva, found in the capture cage, could only have been produced by a single female, or if a captured female larviposited after transfer to a glass tube, but before dissection; and (ii) full-term pregnant females: those found, on dissection, to have *in utero* a late third instar larva of a size indicating that more than 95% of the pregnancy was complete [19]. Flies with empty uteri, which were not seen to have produced a pupa in the burrow or while in a glass tube, were classified as having aborted, or having produced an offspring prematurely, if the largest oocyte had not achieved a mature length of 1.60 mm. These flies, along with all males, females in ovarian category 0, and those that were pregnant but had not completed 95% of pregnancy, were excluded from this analysis.

### 2.2. Age estimation through ovarian dissection

Adult females were dissected to score ovarian category as a measure of age. Flies were dissected, in a random order, as soon as possible after transfer to the laboratory: more than 95% of captured flies were processed on the day of capture, and within approximately 4 h of capture. We did not delay dissection to allow a female to complete larviposition. Ovarian dissection was conducted following the method of Challier [31], described by Hargrove [32]. The relative sizes of the oocytes within the ovarioles in the left and right ovaries were used to establish the number of times the female had ovulated. Ovarian category 0 flies had an empty uterus and had not ovulated for the first time. This ovulation occurs at the age of approximately 8 days [32], and larvae are deposited thereafter at intervals of approximately 9 days. Females that have just deposited their *n*th larva will thus be approximately 8 + 9*n* days old. Published studies indicate that the oocyte and uterine inclusions grow in a highly predictable manner such that it is possible to use their lengths to estimate chronological age to within a few days [27,33–35]. In this study, we do not require this level of precision: we only need to know the number of times a female has ovulated—i.e. its age to the nearest 9 days. The exact number of ovulations completed can, however, only be judged for flies that have ovulated fewer than four times. Thereafter, the ovarian category can only be defined *modulo* 4 [31].

### 2.3. Age estimation using wing fray analysis

Given the above limitation, we also assessed the degree of wing fray in all flies sampled. Heads and legs were removed prior to dissection and discarded: excised wings were appended with transparent tape to the dissection record form for assessment of wing length, as an index of maternal size. Wing fray category, measured on a scale of 1 (perfect wing) to 6 (wing with large rounded indentations), was assessed to provide an approximate and relative measure of fly age [36]. This measure is not as accurate as that derived through ovarian dissection, but is not subject to the limitation referred to above. Wing fray also provides a measure of the stress experienced by a fly. Females with badly frayed wings—regardless of their chronological age—should be at a disadvantage because of their reduced flight performance. They will be less effective in locating hosts and, having fed, will be less able to evade predators when carrying a heavy larva, or a blood meal that can increase their body weight by a factor of more than three [37,38].

### 2.4. Estimating relationships between maternal age and offspring size

In estimating this relationship, one would like, ideally, to collect series of pupae produced by the same fly. However, as all females were dissected on capture, we only ever saw one pupa from each individual female. Accordingly, we can only compare the sizes of pupae produced by different flies, of different ages, at their first, second, third etc. attempt. The effect of age is adjusted for maternal condition and the environmental factors experienced by the female during pregnancy.

### 2.5. Nutritional analysis

All dissected flies were analysed for their nutritional levels, as fully described by Hargrove & Muzari [19]. Adult female thoraces and abdomens were placed in numbered wells of plastic immunoassay plates, and any third instar (L3) larvae or pupae in a separate well. They were dried and stored over calcium chloride and later transferred to individual pre-weighed glass tubes, desiccated in an oven at 70°C over silica gel, and the tube plus fly reweighed to give the dry weight (DW) of the fly, or L3 larva or pupa. All weights were assessed using a Mettler AT-201 electronic balance (Mettler-Toledo, Greifensee, 181 Switzerland) with a precision of 0.01 mg. Fat was extracted in three changes of chloroform over 72 h, the flies re-dried and reweighed to get the residual dry weight (RDW) and, by subtraction, fat contents of adult fly or offspring (in larval or pupal form). The adult thorax was weighed to estimate thoracic residual dry weight (TRDW), and the abdominal residual dry weight (ARDW) was estimated by difference.

Haematin, the iron-containing remnant of haemoglobin that provides an indication of the time since a fly last fed, was measured as detailed by Hargrove [39]: the dry fat-free abdomen was ground in a centrifuge tube; 2.5 ml of 3:2 v:v 0.1 M sodium hydroxide and 80% ethanol was added, mixed, left to stand for 1 h, mixed again and centrifuged at ≈500 *g* for 10 min. The supernatant was decanted into a clean tube containing 0.5 ml pyridine and 30 ± 5 mg of sodium dithionite. After mixing, the pink colour (due to pyridine haemochromogen) was first assessed by eye. If the colour was weak, an aliquot of the sample was transferred to a cuvette and the absorbance measured at 417 nm. Absorbance increased linearly between 0 and 10 mg of haematin; thereafter the response was nonlinear. Accordingly, if there was a deep pink colour, the sample was read at 558 nm, at which wavelength the response increased linearly over the range 0–100 mg of haematin. For higher haematin contents, the sample was diluted by the addition of a 3:2:1 v:v:v mixture of 0.1 M sodium hydroxide, 80% ethanol and pyridine. Standard curves were prepared at each wavelength and both gave comparable results. Using 417 nm produced greater accuracy for haematin less than 10 mg: for higher haematin contents, readings at 558 nm were more convenient and gave acceptable levels of accuracy. Only 20 samples were processed at a time, so all could be read within 10 min of the addition of pyridine, ensuring no significant fading of colour. Absorbency values were converted into haematin content using the standard curves, taking into account any dilution made.

### 2.6. Statistical procedures

Regression analyses were carried out using the Stata statistical package, v. 12 [40]. We used stepwise multiple linear regression to investigate age-related changes in offspring size (measured as dry weight, hereafter termed ‘puparial dry weight’ although this refers both to the dry weight of pupae and of L3 larvae). Age-related changes in puparial dry weight were adjusted for the mother's size, as indicated by the residual dry weight of her thorax (TRDW), and post-partum fat and haematin contents, and for the mean temperature (*T*_9_) over the nine days prior to the day of larviposition, which reflects the approximate duration of the pregnancy giving rise to the pupa in question [35]. We also ran the stepwise multiple linear regression using ovarian category as a categorical variable, so that adjusted values of puparial dry weight could be calculated for each category, using the appropriate covariance matrix, for mothers from each ovarian category. We examined the strength of effects in terms of model coefficients and associated confidence intervals, and denote statistical significance at the 0.05, 0.01 and 0.001 levels of probability by *, ** and ***, respectively: *p *> 0.05 is denoted by ‘ns’.

## 3. Results

A total of 2638 female *G*. *pallidipes* were captured from the burrows during the study and successfully classified according to their pregnancy status, resulting in 927 perinatal flies (i.e. caught immediately before or after giving birth) which were the subject of this study. We provide further details on the month of capture and age structure of flies caught in the electronic supplementary material (electronic supplementary material, figure S1 and table S1). We also use previously published data for *G. m. morsitans* females captured during the same experimental procedure [20].

### 3.1. Fly size as a function of age

**Figure 1.**
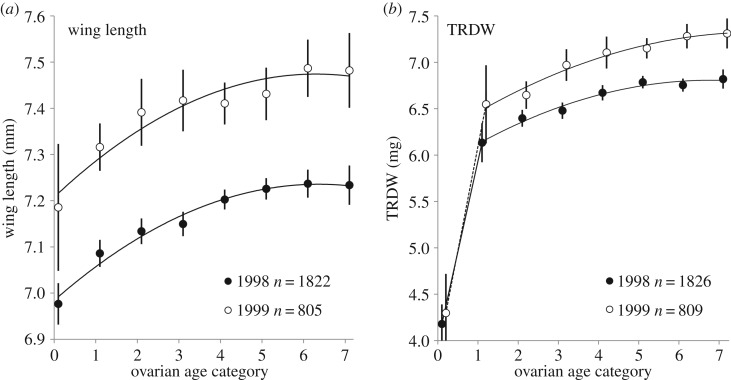
Maternal size as a function of ovarian age. (*a*) Wing length. (*b*) Thoracic residual dry weight (TRDW). *Glossina pallidipes* females captured in artificial warthog burrows: Rekomitjie Research Station, September to November 1998 and 1999. Error bars denote 95% confidence intervals.

Older flies captured in this study had longer wings (regression of wing length on ovarian category between 0 and 7, *R*^2^ = 0.98 in 1998, 0.92 in 1999, *p *< 0.001; figure 1*a*), despite the fact that the linear dimensions of an individual adult tsetse fly do not change with age. The result is due to the fact that the mean wing length of a teneral fly emerging from a puparium varies with season [28,39]. Flies emerging at the hottest time of the year, in mid-October, tend to be small: by contrast, a fly which is in ovarian category 7 at this time emerged at least 2 months previously, and was deposited at least 3 months previously—i.e. in mid-July—at the coolest time of the year. Pupae deposited at this time, and the teneral adults emerging from those pupae, are larger [28].

Thoracic residual dry weight (TRDW) can also be used as an index of size, but TRDW does increase during the first week of life [28,39]. Thereafter, increases with age in TRDW (linear regression of TRDW and ovarian category between 1 and 7, *R*^2^ = 0.98 in 1998, 0.97 in 1999, *p *< 0.001; figure 1*b*) reflect the larger physical dimensions of flies emerging in cooler months of the year, which are the older flies in this study, as explained above.

### 3.2. Offspring dry weights as a function of maternal characteristics

**Figure 2.**
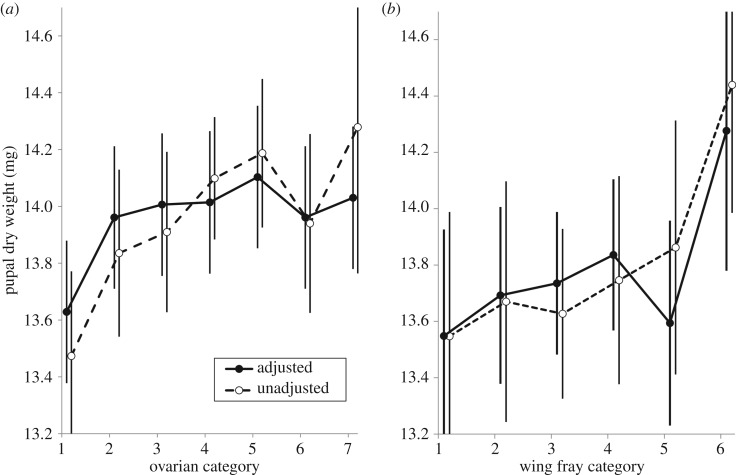
Puparial dry weight as a function of maternal ovarian age (*a*) or wing fray (*b*). Analysis based on 750 *G. pallidipes* females captured in artificial warthog burrows at Rekomitjie Research Station, September to November 1998 and 1999. Age-specific weights are plotted either as unadjusted values, or adjusted for maternal TRDW, fat and haematin levels and also for the mean temperature on the 9 days prior to larviposition. Error bars denote 95% confidence intervals.

Multiple linear regression on puparial dry weight showed a positive linear effect of ovarian category, included originally as a continuous variable (table 1). Note, however, that this effect was weak and sensitive to the exclusion of outliers (electronic supplementary material, figure S2). When ovarian category was included as a categorical variable, puparial dry weight increased over the first five pregnancies (figure 2*a*), but the major age effect was limited to the difference between the dry weight of the first pupa and the dry weight of all later pupae. When the analyses were rerun excluding data for the first pupa, there was no effect of ovarian age on puparial dry weight. Puparial dry weight also increased with the TRDW of the mother, and with her post-partum fat content (table 1). Conversely, puparial dry weight decreased with increasing haematin content and with mean temperature over the nine days of pregnancy (*T*_9_). There was no significant difference between the dry weights of larvae deposited by flies prior to dissection and those of full-term late third instar larvae. When wing length was used instead of TRDW as a measure of fly size, the size effect just failed to achieve statistical significance at the 0.05 level (*p* = 0.054). While pupae produced in 1999 were heavier than those produced in 1998, this effect was due to the larger size of the mothers in that year: once size was included in the model, there was no additional effect of year of capture.

**Table 1.** Multiple linear regression of tsetse puparial dry weight on maternal ovarian age or wing fray category. The dependent variable is the dry weight, in mg, of the puparium, either deposited by the female after capture, or dissected from the uterus as a full-term third instar larva. *Glossina pallidipes* captured in burrows, Rekomitjie Research Station, September–November 1998 and 1999. Ovarian category: *R*^2^* *= 0.29; *N* = 750. Wing fray: *R*^2^* *= 0.29; *N* = 746. Only those terms with a significant effect are included in the table. *p < 0.05, **p < 0.01, ***p < 0.001, ns *p* > 0.05.

	ovarian age			wing fray		
		95% confidence interval			95% confidence interval	
variable	coefficient	low	high	coefficient	low	high
age (linear)	0.0559	0.002	0.110**	0.088	0.022	0.154**
TRDW (mg)	0.338	0.211	0.465***	0.342	0.212	0.472***
fat (mg)	0.334	0.245	0.423***	0.358	0.268	0.448***
log_10_haem (ng)	−0.686	−0.884	−0.487***	−0.696	−0.895	−0.497***
temperature (*T*_9_)	−0.254	−0.307	−0.201***	−0.258	−0.311	−0.205***
constant	21.7	17.8	21.7***	19.8	17.8	21.8***

When we used wing fray instead of ovarian category as a measure of age, puparial dry weight also increased linearly with wing fray, having adjusted for significant effects of maternal TRDW, fat and haematin values, and for mean temperature over the 9 days preceding larviposition (table 1). There was no quadratic effect of wing fray. There was little change in pupal dry weight for flies in wing fray categories 1–5 (figure 2*b*), but the point estimate of the dry weight of pupae produced by females in wing fray category 6 was 0.8 mg higher than for those produced by flies in wing fray category 1 (*p *< 0.05 for the difference). When flies in wing fray category 6 were excluded from the analysis, there was no effect of wing fray on pupal size.

### 3.3. Offspring dry weights as a function of temperature—species difference

To compare the effect of increasing temperature during pregnancy on pupal size in the two species, we applied the full model (table 1) to the *G. m. morsitans* data. Using the regression coefficients, and the mean values for all of the independent variables, we predicted pupal weight for 9-day running mean temperatures between 22.4 and 31.6°C, the extremes observed during the experiment.

Over this temperature range, predicted pupal dry weight for *G. m. morsitans* drops by 0.9 mg, from 9.5 mg to 8.6 mg—a decrease of 9.8%. For *G. pallidipes* the pupal dry weight drops by 2.4 mg, from 15.2 to 12.9 mg—a decrease of 15.3%. As *G. pallidipes* is bigger than *G. m. morsitans*, the bigger absolute decline in pupal weight is not unexpected: the more interesting result is that the proportional decline is 56% greater than the decrease seen in *G. m. morsitans*.

### 3.4. Maternal post-partum fat levels

The mother's post-partum fat levels increased both with maternal size, as measured by her TRDW, and with increasing haematin levels (table 2). Post-partum females had 0.4 mg more fat in 1999 than in 1998. Flies that had already larviposited by the time they were dissected had 0.3 mg less fat than flies full-term pregnant at dissection—in accord with the observation that fat is still being rapidly transferred from mother to larva during the last stages of pregnancy [19]. Post-partum fat levels did not decrease significantly with ovarian age, but there was a highly significant decline with increasing wing fray (table 2).

**Table 2.** Multiple linear regression of post-partum maternal fat levels on ovarian age, or wing fray category, and other factors. The dependent variable is the fat level (in mg) of the mother after she has either deposited a pupa or produced a full-term third instar larva. *Glossina pallidipes* captured in burrows, Rekomitjie Research Station, September–November 1998 and 1999. *R*^2^* *= 0.13; *N* = 745. *p < 0.05, **p < 0.01, ***p < 0.001, ns *p* > 0.05.

	ovarian age			wing fray		
		95% confidence interval			95% confidence interval	
variable	coefficient	low	high	coefficient	low	high
age (linear)	−0.020	−0.063	−0.023 ns	−0.122	−0.171	−0.074***
TRDW (mg)	0.252	0.136	0.368***	0.270	0.173	0.366***
log_10_haem (ng)	0.180	0.019	0.341*	0.283	0.137	0.430***
pupa versus L3 larva	−0.350	−0.511	−0.189***	−0.357	−0.513	−0.202***
temperature (*T*_9_)	−0.058	−0.102	−0.014*	−0.045	−0.086	−0.005*
year (1999 versus 1998)	0.349	0.180	0.517***	0.366	0.206	0.525***
constant	2.81	1.09	4.53**	2.26	0.743	3.77**

### 3.5. Win-win? Females producing the largest pupae have most fat post-partum

In the above analyses, the larger the pupa that a female produces, the more fat she has left, on average, after she has produced that pupa. Regressions of pupal fat content (FatP), estimated as 32% of the pupal dry weight [39], against maternal post-partum fat levels (FatM) are shown in electronic supplementary material, figure S3. Results are shown for the present *G. pallidipes* data and also for previously published data for *G. m. morsitans* [20].

**Figure 3.**
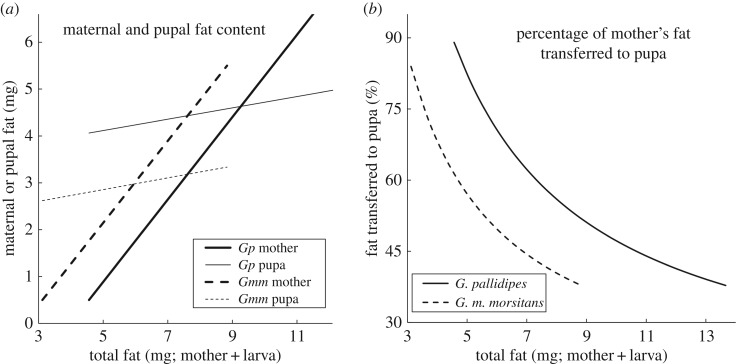
(*a*) Predicted fat content of female *G. pallidipes* and *G. m. morsitans* and of their pupae at full-term pregnancy, plotted against the total fat of mother plus pupae. (*b*) Predicted percentage of the total fat in a full-term pregnant female that is found in the third instar larva immediately before larviposition. Analysis based on 359 *G. m. morsitans* and 754 *G. pallidipes* captured in artificial warthog burrows at Rekomitjie Research Station, September to November 1998 and 1999. Lines are predicted values obtained using the regression equations FatP = 2.55 + 0.143 × FatM (*G. m. morsitans*) and FatP = 3.99 + 0.136 × FatM (*G. pallidipes*).

For 359 *G. m. morsitans*, the function was FatP = 2.55 + FatM × 0.143 (*R*^2^ = 0.093) and for 754 *G. pallidipes*, FatP = 3.99 + FatM × 0.136 (*R*^2^ = 0.124). We used these relationships to calculate, by summation, the total fat content (mother plus larva) in a full-term pregnant fly. Figure 3*a* shows that, as expected, the higher the total fat content at full-term pregnancy, the greater is the amount of the mother's fat that is transferred to the full-term larva. The mother's own fat content also increases with increasing total fat, however, and her own fat content rises more steeply than that of her pupa. The net effect is that the higher the total fat content, the smaller is the *proportion* of fat that is transferred to the pupa (figure 3*a*) so that the flies with the highest total fat can produce the biggest pupae and still have the largest amount of fat post-partum. The relationships between pupal and total fat contents were qualitatively similar for both species—with the graphs for *G. pallidipes* simply shifted up on each axis, in accord with the larger size of this species (figure 3*a*). The percentage of fat transferred to the pupa, accordingly, was similar for both species (figure 3*b*).

## 4. Discussion

Our results indicate that larger female *G. pallidipes,* and those that have experienced cooler temperatures during pregnancy, produce larger pupae. We find only weak evidence that investment changes with age. These results are similar to those published previously for *G. m. morsitans* [20]. We show, however, that even though absolute investment increases with female condition in both species, the relative amount of fat invested declines: females with more fat transfer proportionately less of their fat to their offspring. Consequently, larger females produce larger offspring but retain more reserves post-partum. We discuss below the implications of our results both for understanding life-history patterns and for improved modelling of the population dynamics of this important disease vector.

In *G*. *m*. *morsitans*, a laboratory study found no effect of a mother's age on the size of offspring she produced [41]. A field study reached a similar conclusion: age effects were small, although it was suggested that the youngest mothers produced smaller offspring than all older flies [20]. Here, we find that, for *G*. *pallidipes*, offspring size changes with maternal age, estimated using either ovarian dissection or wing fray analysis. The absolute changes in pupal size with ovarian age are, however, only of the order of 1 mg between the smallest and largest mean sizes—i.e. 7% of the adjusted mean dry weight (14.1 mg) of the largest pupae, produced by flies in ovarian category 5. Moreover, as found by English *et al.* [20], this difference reflects mainly the small size of the first pupa. For later pupae, the mean adjusted pupal dry weights all lie between 14.0 and 14.1 mg. Similarly, for flies in wing fray categories 1–5, the range of pupal dry weights is only 0.3 mg, and for those in category 6, the mean pupal dry weight is only 0.8 mg higher than that for those in category 1. Further work is required to understand why females produce smaller offspring in their first reproductive attempt in both *G. pallidipes* and *G. m. morsitans*. It may be that young females have yet to take on enough blood meals and thus lack the reserves required to produce a large offspring, or that females need to go through one reproductive cycle for all the physiological machinery to be fully primed for reproduction. Both possibilities fit with existing explanations for the relationship between maternal and offspring size [42,43], where theory and data based on turtles and plants, respectively, have shown that morphological constraints of the reproductive tract and efficiency of resource transfer affect the size offspring produced.

The fact that we do not find any evidence that reproductive investment declines with age supports findings in laboratory tsetse that reproductive senescence appears to be absent in this species ([41], but see [44]). It is quite possible, however, that—in contrast to laboratory flies—females in natural conditions do not live long enough to exhibit signs of senescence. In the wild, flies rarely survive beyond 90 days, while age-related declines in fecundity of laboratory flies are only evident after around 120 days [44]. If it is true that females do not exhibit reproductive senescence, this sets tsetse apart from many other vertebrate and invertebrate species that do show reproductive senescence in the wild (reviewed in [45]), which is surprising given the likely high costs of reproduction in this viviparous fly. Such lack of senescence may be due to mechanisms having evolved to mitigate such costs: it has recently been shown that female *G. m. morsitans* increase the expression of antioxidant enzymes during reproduction [46], thus avoiding oxidative costs of reproduction. Indeed, such a finding raises the possibility that tsetse exhibit ‘oxidative shielding’, whereby breeding females have evolved mechanisms to reduce levels of oxidative damage during pregnancy at a time when exposure to such damage may be particularly harmful to offspring [47]. Evidence for such shielding has so far been demonstrated in mammals and birds: viviparous invertebrates such as tsetse present an intriguing test model to establish whether this phenomenon extends to other taxa.

The amount of fat in a female tsetse, just after she has larviposited, provides another measure of maternal investment and, here again, we found no change with ovarian category in these levels. When wing fray was used as a measure of age, however, we found that post-partum fat levels decreased significantly in *G. pallidipes* females with more frayed wings. The different relationships between post-partum fat and age, as measured by ovarian age and wing fray, are not necessarily inconsistent. While there is a high correlation between ovarian age and wing fray, the correlation is not perfect. Conditions experienced by individual flies, and chance events, will inevitably mean that flies of the same chronological age suffer different levels of wing fray. Our results are consistent with the idea that the degree of wing fray is a better index than chronological age of the stress suffered by the female and that this stress is reflected in her post-partum fat levels. Nonetheless, further work is required to tease out the various effects of age, *per se*, and wing fray on maternal condition and on absolute and relative levels of maternal investment.

It may be objected that the lower fat levels in post-partum females with badly frayed wings could reflect increased activity in the capture cages, resulting both in badly frayed wings and in reduced fat levels. Our analyses showed, however, that wing fray for flies at any given ovarian age was lower for flies captured in burrows than for those captured in traps or even on electric nets (electronic supplementary material, figure S4). These results are consistent with the idea that relatively little wing damage occurred in the burrow capture cages. By the same terms, the apparently low levels of wing damage suggest that the flies were not excessively active in the cages and should therefore have used little fat. Flies with severely frayed wings and, thereby, reduced flight capability, may be unable to feed efficiently and safely during gestation and to replenish the fat reserves they transferred to their offspring. It is also possible that females in poor condition, and with reduced chances of survival, invest relatively more in current reproduction. In this sense, tsetse join the ranks of other invertebrates (e.g. [12,13]) and vertebrates (e.g. [48]) in supporting terminal investment theory [16], whereby relative investment into offspring should increase as the intrinsic risk of death for females increases. In contrast to other studies, however, it is not in the measure of chronological age or absolute investment towards offspring that such effects are apparent, thus emphasizing the importance of measuring physiological wear and tear and *relative* reproductive allocation when testing terminal investment theory.

In other respects, maternal physiological state and temperatures experienced during gestation have strong influences on offspring size at birth. Our results reflect those of our earlier study [20] and add to the growing body of empirical findings that larger mothers tend to produce larger offspring [21]. As we discuss in our earlier paper, this pervasive pattern seems to be driven—at least in tsetse—by an underlying relationship between maternal size and body condition: females that can afford to invest more in their offspring do so, but at a lower cost to their own future reproduction. This is because larger females invest absolutely more resources in their offspring but, as we show here, they retain a proportionately higher amount of resources for themselves. We explain this result in the context of patterns of directional selection for offspring size as follows. Classical work on field tsetse populations has shown that very small neonatal flies (at least males) have a lower survival probability than larger neonatal flies, particularly under hot conditions [49]. There is thus strong selection against producing very small offspring, i.e. against depositing undersized larvae or pupae, which may result in a minimum threshold offspring size that all females should aim to produce. That being the case, smaller or less-well-nourished females will need to use a greater proportion of their reserves to ensure that the pupa they produce is greater than the minimum size. These smaller females will, however, endanger their own survival if they transfer a larger proportion of reserves than the minimum necessary for offspring survival. Thus, in tsetse, the explanation for larger females producing larger offspring may simply be because larger females can afford to transfer these reserves without jeopardizing their own chances of surviving to breed again. Equally, if it is true that there is a size of these adult flies above which individuals have relatively lower survival probability, then mothers should not produce pupae so large that they give rise to adults larger than this limit.

Two additional aspects of tsetse biology can help explain such optimal shunting of reserves into offspring depending on maternal state. First, unlike other viviparous species, e.g. mammals [50] and fish [51], female tsetse delay transfer of fat and protein to the developing offspring until late in gestation [19]. Such a strategy may be adaptive if females can delay investing in their costly offspring until they are certain of producing an offspring above the minimum threshold size. Before this point, they can simply abort their pregnancy and—if they have not yet invested much in their larva—this will be at minimal cost. Second, owing to their high-nutrition diet of blood, tsetse exhibit an income breeding strategy [52]. This means that females may not incur long-term nutritional costs of reproduction as they can take a blood meal shortly after giving birth, before embarking on producing their next larvae, and will then take at least another two meals during gestation. It therefore makes sense for females to invest proportionately more fat into their offspring, even if they do not have high fat reserves themselves, to ensure offspring survival in the face of strong selection against small size, as adult females can replenish these reserves before their next reproductive attempt.

We show that pupal weights decrease significantly with the temperature a mother experiences during pregnancy. The link between developmental temperature (which, in tsetse, is equivalent to maternal gestational temperature) and smaller size of offspring is well established in other ectotherms (reviewed in [23], e.g. [53,54]), probably due to an acceleration of biochemical processes underlying growth, reducing development time and leading to smaller size at maturity [55]. Our results are important in the context of meteorological changes in the neighbourhood of Rekomitjie Research Station. It is becoming increasingly apparent that, in tsetse, pupae and newly emerged tenerals are the most vulnerable stages in the life cycle—and are particularly sensitive to temperature [56]. Over the past 50 years at Rekomitjie Research Station, mean temperatures have increased by nearly 1°C—and these increases have been most severe during November when mean temperatures have risen by more than 2°C [57]. These increases in temperature have been associated with serious declines in the tsetse populations at Rekomitjie, and the reductions have been greater for *G. pallidipes* than for *G. m. morsitans* [57]. The declining sizes of pupae produced as temperatures increase in August through November at Rekomitjie result in smaller emerging adults, which themselves produce even smaller pupae—both because of their own small size and the progressively higher temperatures. Such a relationship is important given much earlier evidence that small teneral flies have dramatically increased mortality during the hottest time of the year [49]. There is thus a positive feedback process during this period that only ends once the rains set in and temperatures drop. Given these exigencies, with continuing temperature increases at Rekomitjie—particularly at the already hottest times of the year—we may expect to see further declines in pupal size and viability during the hot dry seasons in forthcoming years. Our results suggest that these effects will be more severe for *G. pallidipes* than for *G. m. morsitans*, as the rate of decrease in relative pupal weight with increasing temperature is more than 50% higher for *G. pallidipes* than it is for *G. m. morsitans*.

## 5. Conclusion

Our work shows that aspects of female condition and the environment experienced during pregnancy are important in determining the size of offspring produced in viviparous tsetse. In common with previous studies, we found weak effects of maternal age on offspring size: the effects were largely limited to the smaller pupae produced by the youngest mothers and the larger pupae produced by flies that exhibited high physiological wear and tear in terms of wing fray. It has long been established that there is strong selection against small pupae, and our results indicate that mothers strategically invest reserves in their offspring to avoid such mortality costs, even if this comes at a detriment to their own body condition. Consequently, females with lower fat reserves invest proportionately more fat in their offspring. Tsetse thus present an interesting case to test life-history predictions of current versus future reproduction trade-offs. Cross-sectional field studies such as ours should be paired with laboratory experiments, following individuals across several reproductive cycles, to ascertain whether such state-based adjustments serve the long-term fitness interest of females. Given, also, recent indications that the negative effects of high temperature on adult mortality are most severe for the youngest flies [58], studies such as ours provide important inputs for modelling tsetse population dynamics.

## Supplementary Material

Supplementary Material for paper on maternal investment in tsetse

## Data accessibility

All data used in analyses in this manuscript have been archived on: http://dx.doi.org/10.5061/dryad.dk551 [59].
