# Supplementary material for: How maternal investment varies with environmental factors and the age and physiological state of wild tsetse Glossina pallidipes and Glossina morsitans morsitans
Source: R Soc Open Sci. 2018 Feb 14;5(2):171739. doi: 10.1098/rsos.171739 (PMC5830771; doi:10.1098/rsos.171739)
Supplement: Supplementary Material for paper on maternal investment in tsetse [file rsos171739supp1.docx]

**How maternal investment varies with environmental factors and the age and physiological state of wild, viviparous tsetse *Glossina pallidipes* Austen and G. m. *morsitans* Westwood**

John W. Hargrove, M. Odwell Muzari and Sinead English

**Electronic Supplementary Material**

**Details of flies sampled**

Of the 927 perinatal flies (i.e., caught immediately before or after giving birth) 69%, 25% and 6% were captured in September, October and November, respectively. By contrast, for 1711 non-perinatal flies assumed to be entering burrows as a response to high temperatures (Hargrove & Muzari 2015), the percentages in the three months were 11%, 51% and 39%, respectively (see Table S1 for more details).

Among perinatal flies there was a dearth of flies in the first three ovarian categories. In a population with a stable age structure, with even a modest adult mortality of, say, 2% per day we should expect the numbers to decline by a factor of approximately 0.98^9^ = 0.83 with each successive 9-day ovarian category. Instead numbers were approximately constant or even increased slightly between categories 1 and 3 (Figure S1). One might be tempted to conclude from such data that younger flies tend not to deposit their larvae in burrows and that we are seeing a very biased sample of larvipositing flies. That this is not the case is indicated by the fact the age distribution for pre-full-term pregnant flies is very similar (Figure S1). The unusual age distributions are largely due to high mortality at the pupal and teneral stage at this hot dry time of the year (Hargrove & Ackley 2015). The jump in numbers between ovarian categories 3 and 4 is due in part to this effect and in part to the presence in ovarian category 4 of flies that have ovulated 8, 12 *etc*. times. Given, however, that we expect rather small numbers of flies to have ovulated more than seven times, and that numbers keep declining with each extra ovulation, there is still a sharp decline in numbers between ovarian stages 4+4n and 7+4n. This decline accords with the ageing effect identified in laboratory and field tsetse (Jordan & Curtis 1968, 1972; Hargrove, Ouifki & Ameh 2011).

**Figure S1.** Age distributions of perinatal and pre-full-term pregnant *G. pallidipes* captured in artificial warthog burrows: Rekomitjie Research Station, September to November 1998 and 1999. Flies in ovarian category 0 have been excluded from this plot.

**Table S1.** Characteristics of *G. pallidipes* females captured in artificial warthog burrows. Rekomitjie Research Station in 1998 and 1999. The three columns under the heading 1998+1999 give the total numbers of female flies that are perinatal (rows 1–3) or in all other categories (rows 4–6), and the proportions of the totals in each case caught in the different months of the experiment.

|  | 1998 | | | 1999 | | | 1998+1999 | | | Grand  Totals |
| --- | --- | --- | --- | --- | --- | --- | --- | --- | --- | --- |
|  | Sep | Oct | Nov | Sep | Oct | Nov | Sep | Oct | Nov |  |
| 1 Full-term pregnant | 110 | 41 | 1 | 69 | 26 | 20 |  |  |  | 267 |
| 2 Postpartum (pupa) | 281 | 74 | 7 | 146 | 41 | 5 | 69% | 25% | 6% | 554 |
| 3 Postpartum (no pupa) | 24 | 39 | 15 | 11 | 8 | 9 | 641 | 229 | 57 | 106 |
| 4 Ovarian category 0 | 2 | 61 | 42 | 5 | 7 | 21 |  |  |  | 138 |
| 5 Abortions/premature | 16 | 113 | 78 | 15 | 9 | 36 | 11% | 51% | 39% | 267 |
| 6 Pre-full-term pregnant | 59 | 571 | 291 | 83 | 111 | 191 | 180 | 872 | 659 | 1306 |
| Totals | 492 | 899 | 434 | 329 | 202 | 282 |  |  |  | 2638 |

**Outliers in puparial dry weight data**

The distribution of *G. pallidipes* puparial dry weights was skewed to the left, with some obvious outliers on the low side, and none on the high side. With a mean weight of 13.746 mg and standard deviation of 1.851, 2.34 times the standard deviation above the mean gives a value of 18.08 mg, the same weight as the heaviest pupa. If we take the same distance below the mean, and use this as a cut-off for outliers, then we exclude all pupae with dry weight < 9.4 mg. With this cut-off there are significant linear and quadratic effects of age on pupal dry weight, of the same magnitude and direction when the full dataset is considered (see main text). If, however, the cut-off is dropped to 9 mg, the age effects disappear.

**Figure S2.** Distribution of *G. pallidipes* pupal dry weights.

**Maternal and pupal fat**

Figure S3 shows the results of least-squares regressions of pupal fat against maternal postpartum fat for *G. pallidipes* and *G. m. morsitans*. The regression equations are used to predict the pupal fat for mothers with given postpartum fata contents and, thereby, to predict the total fat content of pregnant mother plus full-term larva.

**Figure S3.** Regressions of pupal fat, estimated as 32% of pupal dry weight, regressed against maternal postpartum fat.

**Increase in wing fray with ovarian age, for flies caught using different sampling methods**

We compared the wing fray found in female *G. pallidipes* captured at Rekomitjie Research Station using: (i) odour-baited epsilon traps (Hargrove & Langley, 1990); (ii) a vehicle-mounted electric target (VET; Hargrove, 1990); (iii) the artificial warthog burrows described in this paper.

Traps were cleared every 30 minutes, in an attempt to minimise wing damage subsequent to capture. Nonetheless, it is evident from the results in Figure S4 that these flies show significantly higher mean wing fray, at any given age, than flies caught using the other two methods. Those caught on the VET were killed instantly, and the only excess wing fray would result from the fly’s collision with the net and direct effect of the electric pulses from the screen. These flies show less wing fray than those caught in epsilon traps, but their mean wing fray is marginally higher than for those caught in the burrows.

Notice that in plotting the results in Figure S4, we take cognisance of the fact that all fly that have just produced their first pupa are still, nominally, in ovarian category 1 – but will enter ovarian category 2 within minutes. Conversely, flies in ovarian category 1, when captured in traps or on the VET, are distributed across the range of flies that have just ovulated for the first time, to those that are approaching the end of their first pregnancy. The positioning of the points on the ovarian category axis reflects these differences.

**Figure S4.** Mean wing fray found in female *G. pallidipes* as a function of ovarian category and sampling method.

**References**

**Hargrove, J. W.** (1990). Age-dependent changes in the probabilities of survival and capture of the tsetse fly *Glossina morsitans morsitans* Westwood. *Insect Science and its Application* **11,** 323-330.

**Hargrove, J. & Ackley, S. F.** (2015) Mortality estimates from ovarian age distributions of the tsetse fly *Glossina pallidipes* Austen sampled in Zimbabwe suggest the need for new analytical approaches. *Bulletin of Entomological Research* **105**, 294–304.

**Hargrove, J. W. & Muzari, M. O.** (2015) Artificial warthog burrows used to sample adult and immature tsetse (Glossina spp) in the Zambezi Valley of Zimbabwe. *PLoS Neglected Tropical Diseases* **9**, e0003565. (doi:10.1371/journal.pntd.0003565)

**Hargrove, J. W. & Langley, P. A.** (1990) Sterilizing tsetse in the field: a successful field trial. *Bulletin of Entomological Research,* **80**, 397-403.

**Hargrove, J. W. Ouifki, R. & Ameh, J. E.** (2011) A general model for mortality in adult tsetse (*Glossina* spp). *Medical and Veterinary Entomology*, **25**, 385-394.

**Jordan, A. M. & Curtis, C. F.** (1968). Productivity of *Glossina austeni* Newst. maintained on lop-eared rabbits. *Bulletin of Entomological Research* **58**, 399-410.

**Jordan, A. M. & Curtis, C. F.** (1972). Productivity of *Glossina morsitans* Westwood maintained in the laboratory, with particular reference to the sterile - insect release method. *Bulletin of the World Health Organisation* **46**, 33-38.
